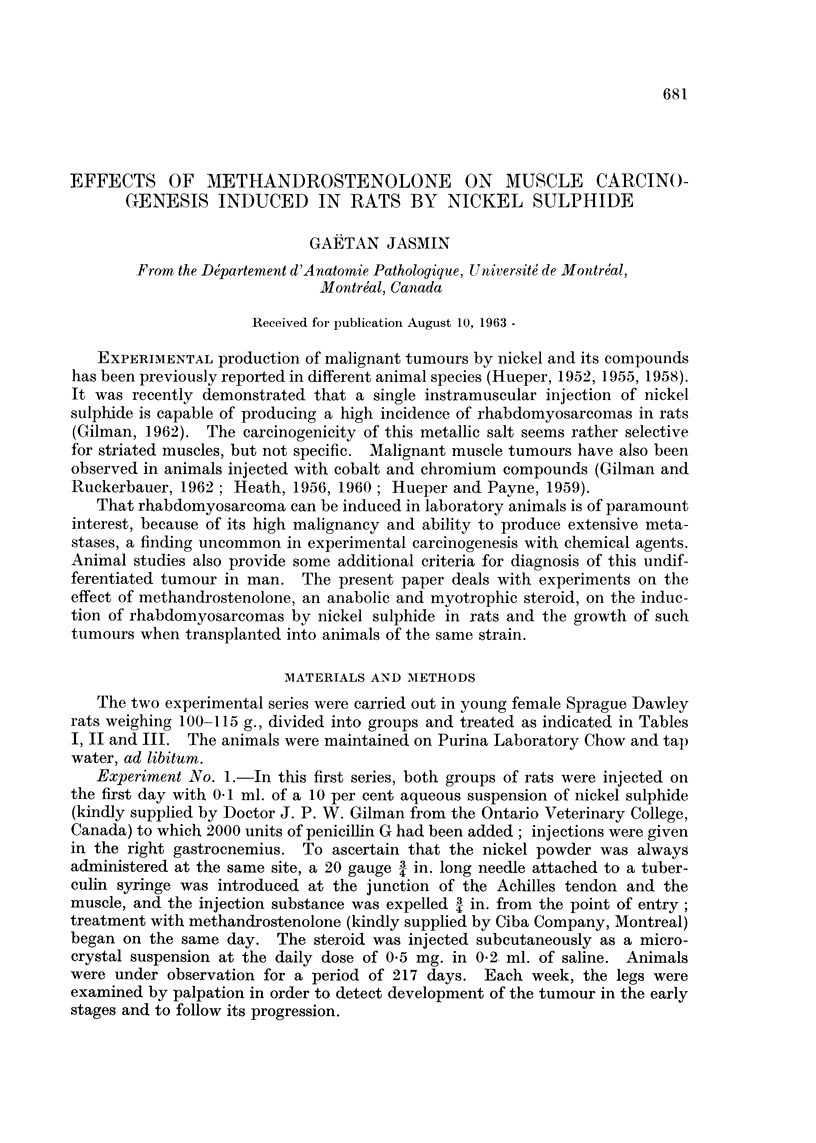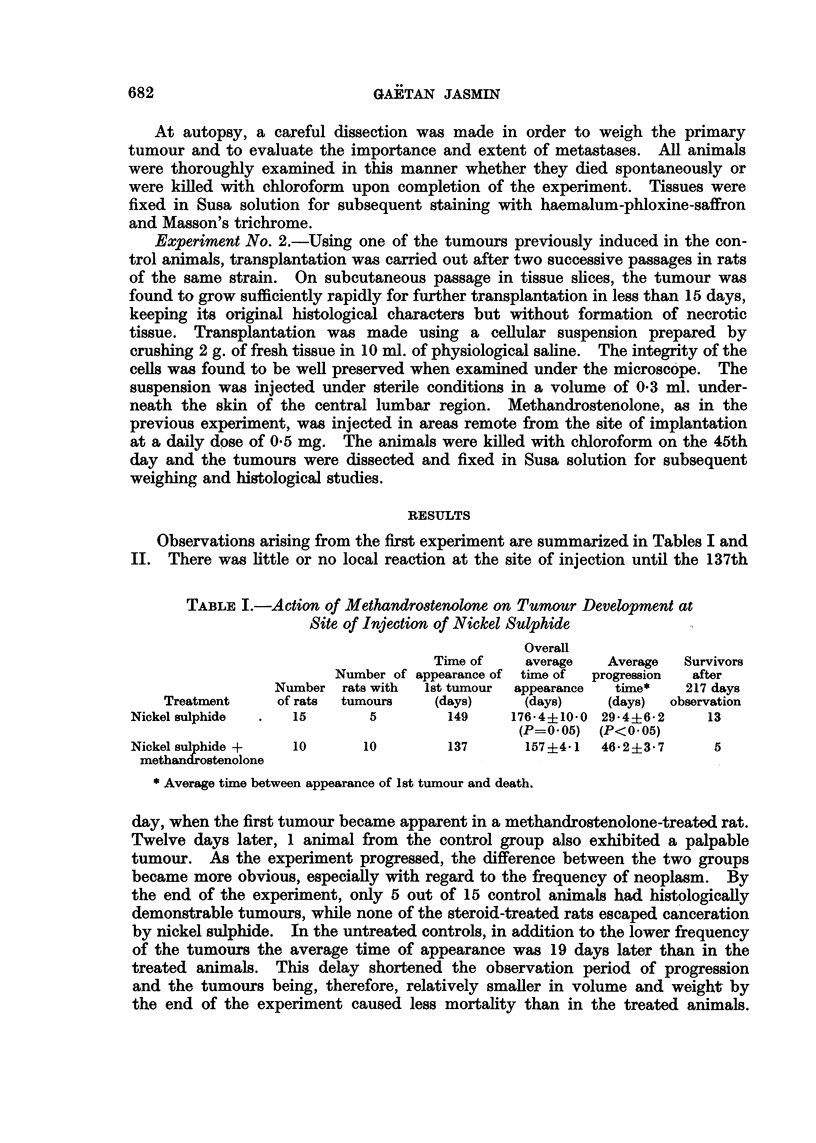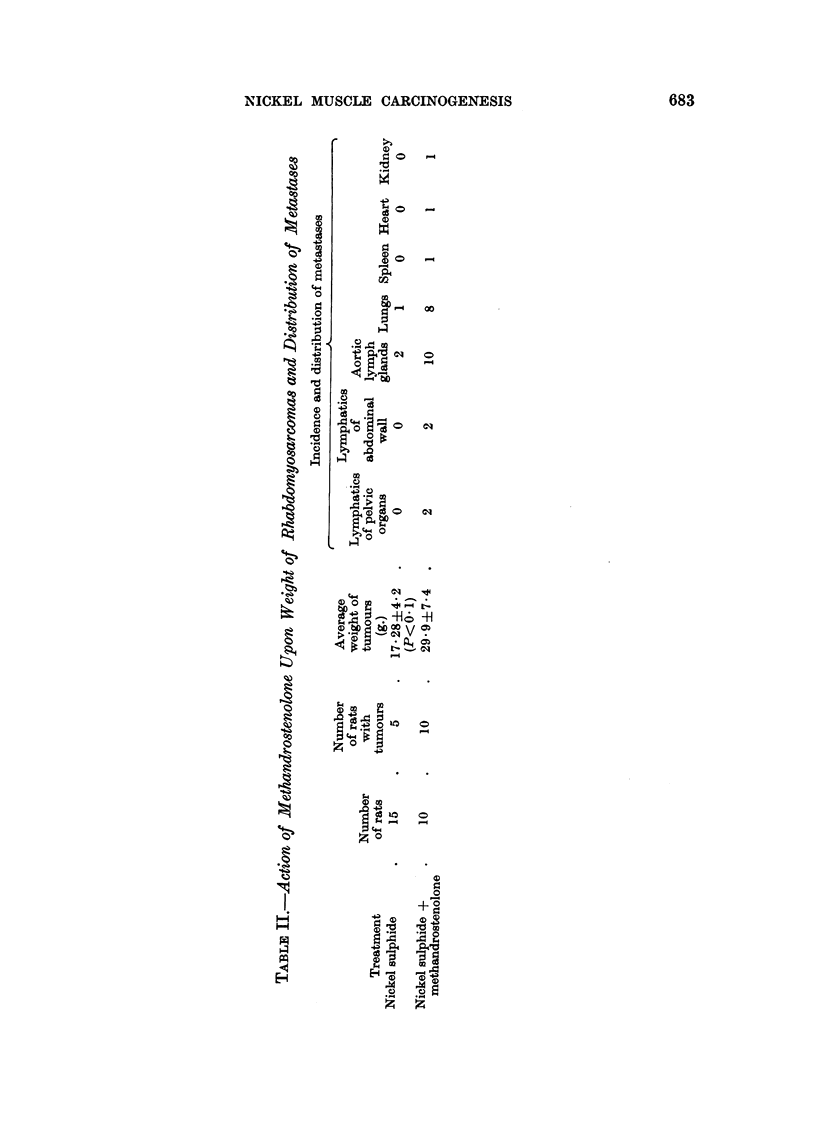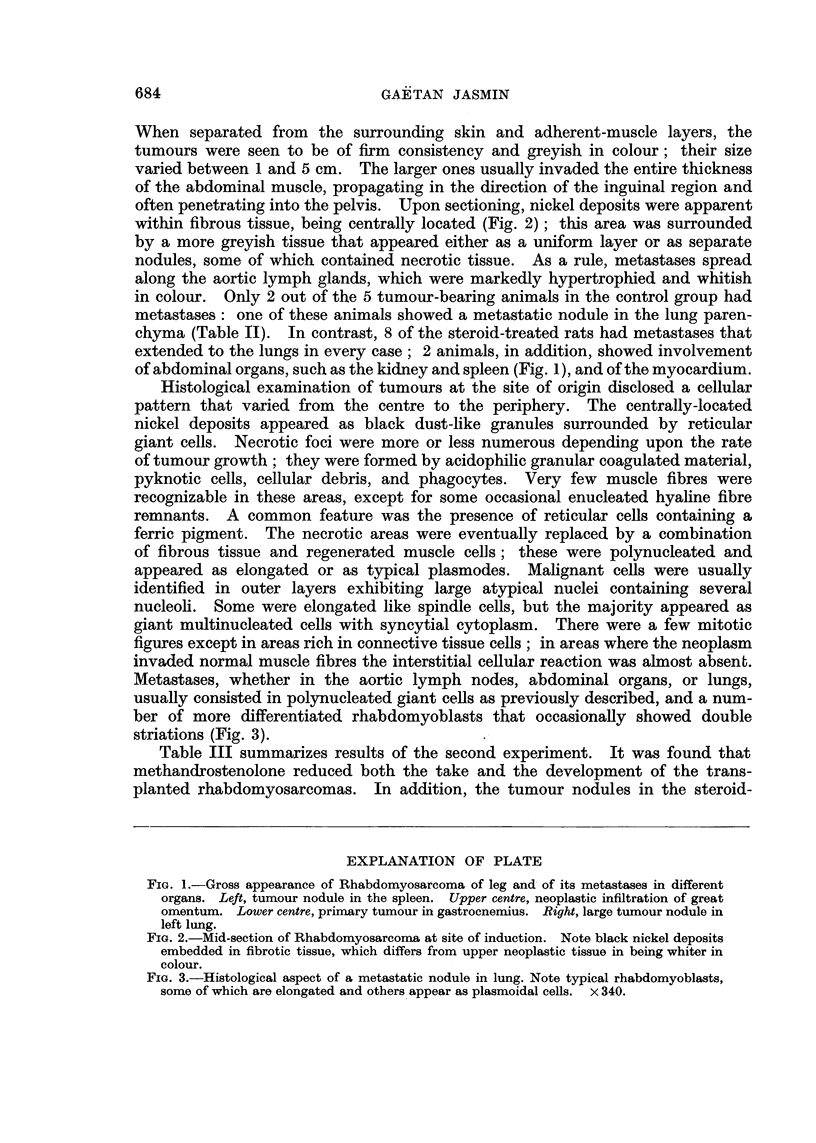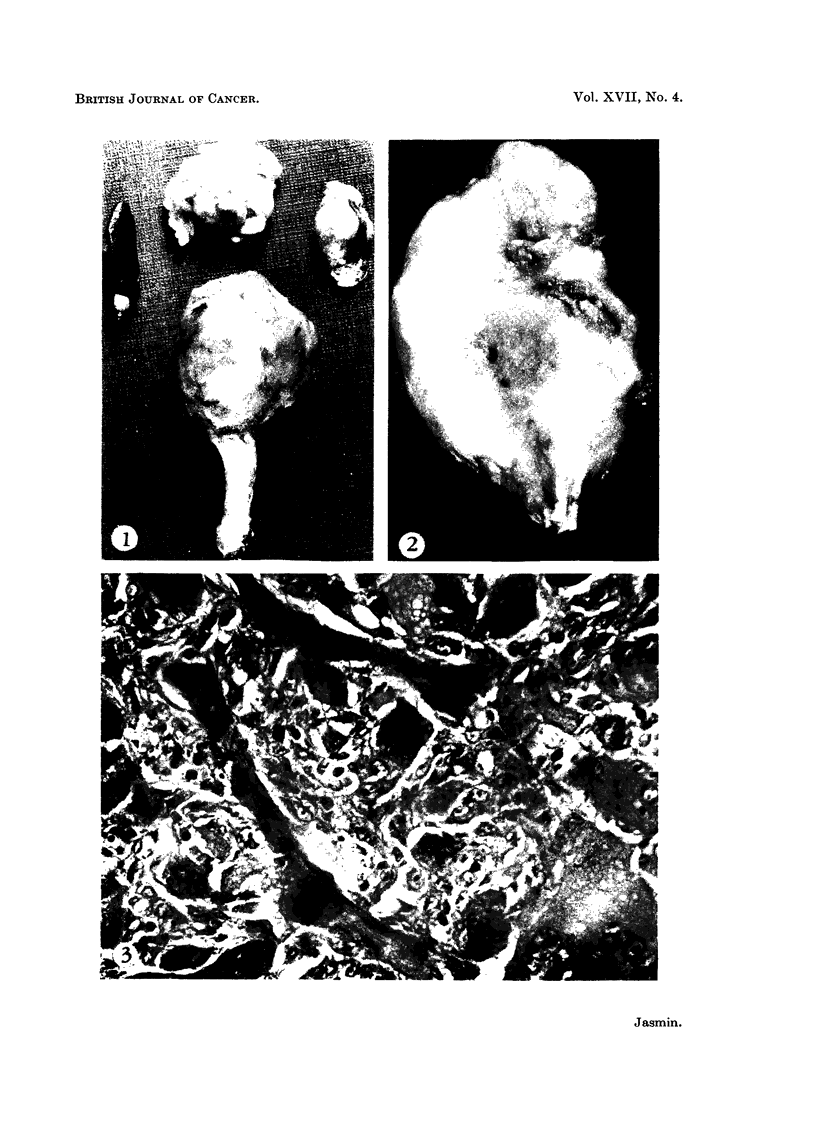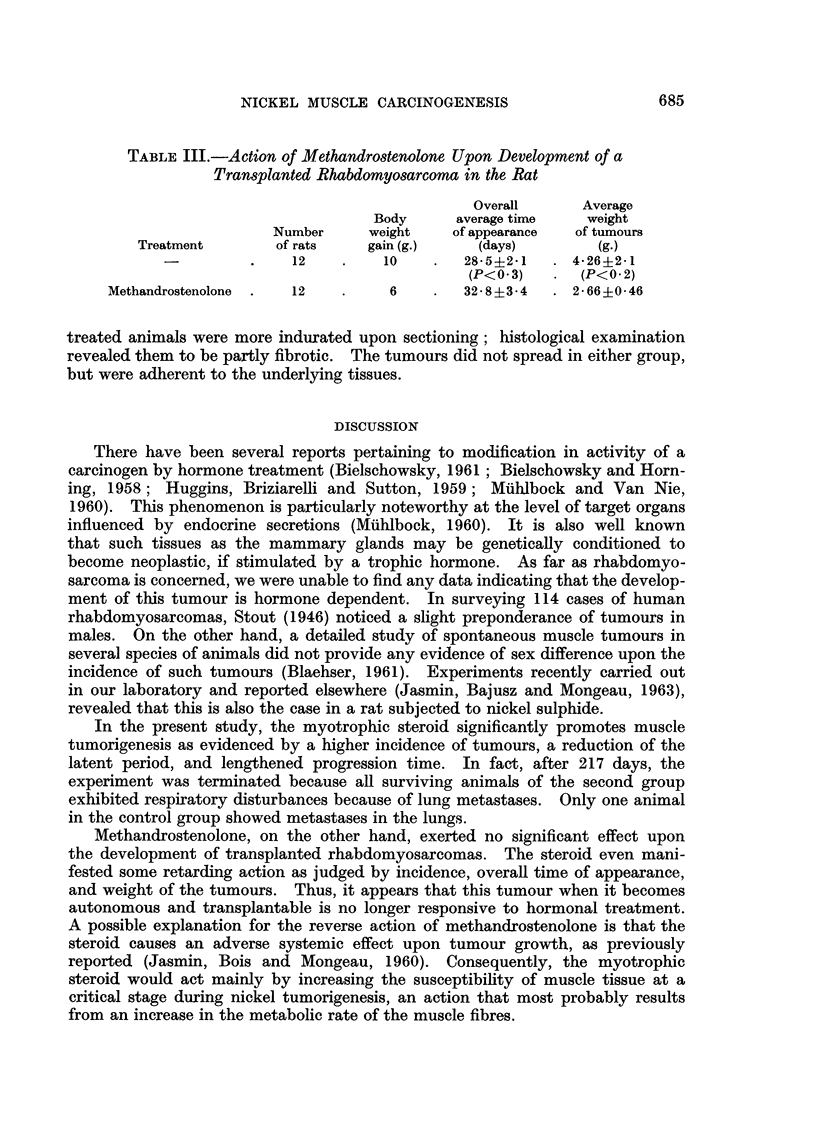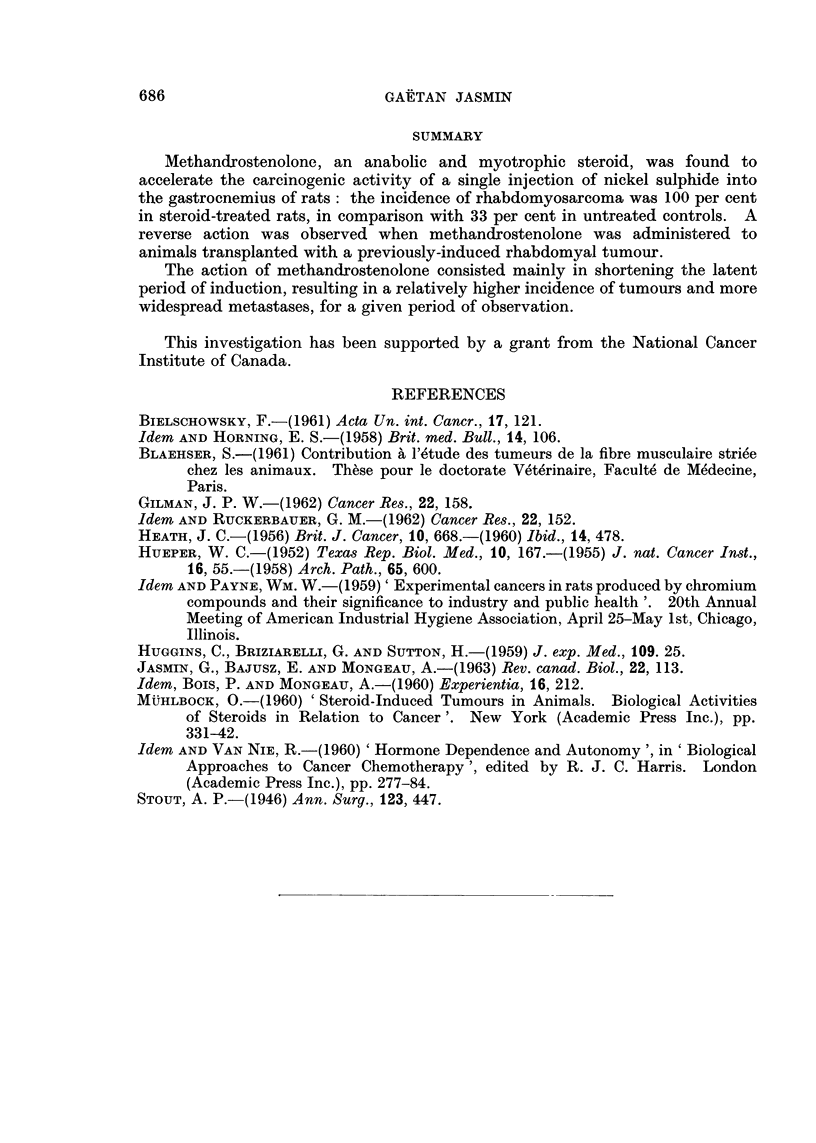# Effects of Methandrostenolone on Muscle Carcinogenesis induced in Rats by Nickel Sulphide

**DOI:** 10.1038/bjc.1963.86

**Published:** 1963-12

**Authors:** Gaëtan Jasmin

## Abstract

**Images:**


### EFFECTS OF METHANDROSTENOLONE ON MUSCLE CARCINOGENESIS INDUCED IN RATS BY NICKEL SULPHIDE

GAËTAN JASMIN

*From the Département d'Anatomie Pathologique, Université de Montréal, Montréal, Canada*

Received for publication August 10, 1963 -

EXPERIMENTAL production of malignant tumours by nickel and its compounds has been previously reported in different animal species (Hueper, 1952, 1955, 1958). It was recently demonstrated that a single instramuscular injection of nickel sulphide is capable of producing a high incidence of rhabdomyosarcomas in rats (Gilman, 1962). The carcinogenicity of this metallic salt seems rather selective for striated muscles, but not specific. Malignant muscle tumours have also been observed in animals injected with cobalt and chromium compounds (Gilman and Ruckerbauer, 1962 ; Heath, 1956, 1960 ; Hueper and Payne, 1959).

That rhabdomyosarcoma can be induced in laboratory animals is of paramount interest, because of its high malignancy and ability to produce extensive metastases, a finding uncommon in experimental carcinogenesis with chemical agents. Animal studies also provide some additional criteria for diagnosis of this undifferentiated tumour in man. The present paper deals with experiments on the effect of methandrostenolone, an anabolic and myotrophic steroid, on the induction of rhabdomyosarcomas by nickel sulphide in rats and the growth of such tumours when transplanted into animals of the same strain.

### MATERIALS AND METHODS

The two experimental series were carried out in young female Sprague Dawley rats weighing 100–115 g., divided into groups and treated as indicated in Tables I, II and III. The animals were maintained on Purina Laboratory Chow and tap water, *ad libitum.*

*Experiment No.* 1.—In this first series, both groups of rats were injected on the first day with 0·1 ml. of a 10 per cent aqueous suspension of nickel sulphide (kindly supplied by Doctor J. P. W. Gilman from the Ontario Veterinary College, Canada) to which 2000 units of penicillin G had been added ; injections were given in the right gastrocnemius. To ascertain that the nickel powder was always administered at the same site, a 20 gauge $\frac{3}{4}$ in. long needle attached to a tuberculin syringe was introduced at the junction of the Achilles tendon and the muscle, and the injection substance was expelled $\frac{3}{4}$ in. from the point of entry ; treatment with methandrostenolone (kindly supplied by Ciba Company, Montreal) began on the same day. The steroid was injected subcutaneously as a microcrystal suspension at the daily dose of 0·5 mg. in 0·2 ml. of saline. Animals were under observation for a period of 217 days. Each week, the legs were examined by palpation in order to detect development of the tumour in the early stages and to follow its progression.

At autopsy, a careful dissection was made in order to weigh the primary tumour and to evaluate the importance and extent of metastases. All animals were thoroughly examined in this manner whether they died spontaneously or were killed with chloroform upon completion of the experiment. Tissues were fixed in Susa solution for subsequent staining with haemalum-phloxine-saffron and Masson's trichrome.

*Experiment No.* 2.—Using one of the tumours previously induced in the control animals, transplantation was carried out after two successive passages in rats of the same strain. On subcutaneous passage in tissue slices, the tumour was found to grow sufficiently rapidly for further transplantation in less than 15 days, keeping its original histological characters but without formation of necrotic tissue. Transplantation was made using a cellular suspension prepared by crushing 2 g. of fresh tissue in 10 ml. of physiological saline. The integrity of the cells was found to be well preserved when examined under the microscope. The suspension was injected under sterile conditions in a volume of 0·3 ml. underneath the skin of the central lumbar region. Methandrostenolone, as in the previous experiment, was injected in areas remote from the site of implantation at a daily dose of 0·5 mg. The animals were killed with chloroform on the 45th day and the tumours were dissected and fixed in Susa solution for subsequent weighing and histological studies.

### RESULTS

Observations arising from the first experiment are summarized in Tables I and II. There was little or no local reaction at the site of injection until the 137th

TABLE I.—*Action of Methandrostenolone on Tumour Development at Site of Injection of Nickel Sulphide*

| Treatment | Number of rats | Number of rats with tumours | Time of appearance of 1st tumour (days) | Overall average time of appearance (days) | Average progression time* (days) | Survivors after 217 days observation |
|---|---|---|---|---|---|---|
| Nickel sulphide . | 15 | 5 | 149 | 176·4±10·0 ($P$=0·05) | 29·4±6·2 ($P$<0·05) | 13 |
| Nickel sulphide + methandrostenolone | 10 | 10 | 137 | 157±4·1 | 46·2±3·7 | 5 |

* Average time between appearance of 1st tumour and death.

day, when the first tumour became apparent in a methandrostenolone-treated rat. Twelve days later, 1 animal from the control group also exhibited a palpable tumour. As the experiment progressed, the difference between the two groups became more obvious, especially with regard to the frequency of neoplasm. By the end of the experiment, only 5 out of 15 control animals had histologically demonstrable tumours, while none of the steroid-treated rats escaped canceration by nickel sulphide. In the untreated controls, in addition to the lower frequency of the tumours the average time of appearance was 19 days later than in the treated animals. This delay shortened the observation period of progression and the tumours being, therefore, relatively smaller in volume and weight by the end of the experiment caused less mortality than in the treated animals.

TABLE II.—*Action of Methandrostenolone Upon Weight of Rhabdomyosarcomas and Distribution of Metastases*

| | | | | Incidence and distribution of metastases | | | | | | |
|---|---|---|---|---|---|---|---|---|---|---|
| Treatment | Number of rats | Number of rats with tumours | Average weight of tumours (g.) | Lymphatics of pelvic organs | Lymphatics of abdominal wall | Aortic lymph glands | Lungs | Spleen | Heart | Kidney |
| Nickel sulphide | 15 | 5 | $17 \cdot 28 \pm 4 \cdot 2$ ($P < 0 \cdot 1$) | 0 | 0 | 2 | 1 | 0 | 0 | 0 |
| Nickel sulphide + methandrostenolone | 10 | 10 | $29 \cdot 9 \pm 7 \cdot 4$ | 2 | 2 | 10 | 8 | 1 | 1 | 1 |

When separated from the surrounding skin and adherent-muscle layers, the tumours were seen to be of firm consistency and greyish in colour; their size varied between 1 and 5 cm. The larger ones usually invaded the entire thickness of the abdominal muscle, propagating in the direction of the inguinal region and often penetrating into the pelvis. Upon sectioning, nickel deposits were apparent within fibrous tissue, being centrally located (Fig. 2); this area was surrounded by a more greyish tissue that appeared either as a uniform layer or as separate nodules, some of which contained necrotic tissue. As a rule, metastases spread along the aortic lymph glands, which were markedly hypertrophied and whitish in colour. Only 2 out of the 5 tumour-bearing animals in the control group had metastases: one of these animals showed a metastatic nodule in the lung parenchyma (Table II). In contrast, 8 of the steroid-treated rats had metastases that extended to the lungs in every case; 2 animals, in addition, showed involvement of abdominal organs, such as the kidney and spleen (Fig. 1), and of the myocardium.

Histological examination of tumours at the site of origin disclosed a cellular pattern that varied from the centre to the periphery. The centrally-located nickel deposits appeared as black dust-like granules surrounded by reticular giant cells. Necrotic foci were more or less numerous depending upon the rate of tumour growth; they were formed by acidophilic granular coagulated material, pyknotic cells, cellular debris, and phagocytes. Very few muscle fibres were recognizable in these areas, except for some occasional enucleated hyaline fibre remnants. A common feature was the presence of reticular cells containing a ferric pigment. The necrotic areas were eventually replaced by a combination of fibrous tissue and regenerated muscle cells; these were polynucleated and appeared as elongated or as typical plasmodes. Malignant cells were usually identified in outer layers exhibiting large atypical nuclei containing several nucleoli. Some were elongated like spindle cells, but the majority appeared as giant multinucleated cells with syncytial cytoplasm. There were a few mitotic figures except in areas rich in connective tissue cells; in areas where the neoplasm invaded normal muscle fibres the interstitial cellular reaction was almost absent. Metastases, whether in the aortic lymph nodes, abdominal organs, or lungs, usually consisted in polynucleated giant cells as previously described, and a number of more differentiated rhabdomyoblasts that occasionally showed double striations (Fig. 3).

Table III summarizes results of the second experiment. It was found that methandrostenolone reduced both the take and the development of the transplanted rhabdomyosarcomas. In addition, the tumour nodules in the steroid-

---

EXPLANATION OF PLATE

FIG. 1.—Gross appearance of Rhabdomyosarcoma of leg and of its metastases in different organs. *Left*, tumour nodule in the spleen. *Upper centre*, neoplastic infiltration of great omentum. *Lower centre*, primary tumour in gastrocnemius. *Right*, large tumour nodule in left lung.

FIG. 2.—Mid-section of Rhabdomyosarcoma at site of induction. Note black nickel deposits embedded in fibrotic tissue, which differs from upper neoplastic tissue in being whiter in colour.

FIG. 3.—Histological aspect of a metastatic nodule in lung. Note typical rhabdomyoblasts, some of which are elongated and others appear as plasmoidal cells. ×340.

1

2

3

TABLE III.—*Action of Methandrostenolone Upon Development of a Transplanted Rhabdomyosarcoma in the Rat*

| Treatment | Number of rats | Body weight gain (g.) | Overall average time of appearance (days) | Average weight of tumours (g.) |
|---|---|---|---|---|
| — | 12 | 10 | $28 \cdot 5 \pm 2 \cdot 1$ | $4 \cdot 26 \pm 2 \cdot 1$ |
| | | | $(P < 0 \cdot 3)$ | $(P < 0 \cdot 2)$ |
| Methandrostenolone | 12 | 6 | $32 \cdot 8 \pm 3 \cdot 4$ | $2 \cdot 66 \pm 0 \cdot 46$ |

treated animals were more indurated upon sectioning ; histological examination revealed them to be partly fibrotic. The tumours did not spread in either group, but were adherent to the underlying tissues.

### DISCUSSION

There have been several reports pertaining to modification in activity of a carcinogen by hormone treatment (Bielschowsky, 1961 ; Bielschowsky and Horning, 1958 ; Huggins, Briziarelli and Sutton, 1959 ; Mühlbock and Van Nie, 1960). This phenomenon is particularly noteworthy at the level of target organs influenced by endocrine secretions (Mühlbock, 1960). It is also well known that such tissues as the mammary glands may be genetically conditioned to become neoplastic, if stimulated by a trophic hormone. As far as rhabdomyosarcoma is concerned, we were unable to find any data indicating that the development of this tumour is hormone dependent. In surveying 114 cases of human rhabdomyosarcomas, Stout (1946) noticed a slight preponderance of tumours in males. On the other hand, a detailed study of spontaneous muscle tumours in several species of animals did not provide any evidence of sex difference upon the incidence of such tumours (Blaehser, 1961). Experiments recently carried out in our laboratory and reported elsewhere (Jasmin, Bajusz and Mongeau, 1963), revealed that this is also the case in a rat subjected to nickel sulphide.

In the present study, the myotrophic steroid significantly promotes muscle tumorigenesis as evidenced by a higher incidence of tumours, a reduction of the latent period, and lengthened progression time. In fact, after 217 days, the experiment was terminated because all surviving animals of the second group exhibited respiratory disturbances because of lung metastases. Only one animal in the control group showed metastases in the lungs.

Methandrostenolone, on the other hand, exerted no significant effect upon the development of transplanted rhabdomyosarcomas. The steroid even manifested some retarding action as judged by incidence, overall time of appearance, and weight of the tumours. Thus, it appears that this tumour when it becomes autonomous and transplantable is no longer responsive to hormonal treatment. A possible explanation for the reverse action of methandrostenolone is that the steroid causes an adverse systemic effect upon tumour growth, as previously reported (Jasmin, Bois and Mongeau, 1960). Consequently, the myotrophic steroid would act mainly by increasing the susceptibility of muscle tissue at a critical stage during nickel tumorigenesis, an action that most probably results from an increase in the metabolic rate of the muscle fibres.

SUMMARY

Methandrostenolone, an anabolic and myotrophic steroid, was found to accelerate the carcinogenic activity of a single injection of nickel sulphide into the gastrocnemius of rats : the incidence of rhabdomyosarcoma was 100 per cent in steroid-treated rats, in comparison with 33 per cent in untreated controls. A reverse action was observed when methandrostenolone was administered to animals transplanted with a previously-induced rhabdomyal tumour.

The action of methandrostenolone consisted mainly in shortening the latent period of induction, resulting in a relatively higher incidence of tumours and more widespread metastases, for a given period of observation.

This investigation has been supported by a grant from the National Cancer Institute of Canada.

REFERENCES

BIELSCHOWSKY, F.—(1961) *Acta Un. int. Cancr.*, **17**, 121.
*Idem* AND HORNING, E. S.—(1958) *Brit. med. Bull.*, **14**, 106.
BLAEHSER, S.—(1961) Contribution à l'étude des tumeurs de la fibre musculaire striée chez les animaux. Thèse pour le doctorate Vétérinaire, Faculté de Médecine, Paris.
GILMAN, J. P. W.—(1962) *Cancer Res.*, **22**, 158.
*Idem* AND RUCKERBAUER, G. M.—(1962) *Cancer Res.*, **22**, 152.
HEATH, J. C.—(1956) *Brit. J. Cancer*, **10**, 668.—(1960) *Ibid.*, **14**, 478.
HUEPER, W. C.—(1952) *Texas Rep. Biol. Med.*, **10**, 167.—(1955) *J. nat. Cancer Inst.*, **16**, 55.—(1958) *Arch. Path.*, **65**, 600.
*Idem* AND PAYNE, WM. W.—(1959) ' Experimental cancers in rats produced by chromium compounds and their significance to industry and public health '. 20th Annual Meeting of American Industrial Hygiene Association, April 25–May 1st, Chicago, Illinois.
HUGGINS, C., BRIZIARELLI, G. AND SUTTON, H.—(1959) *J. exp. Med.*, **109**. 25.
JASMIN, G., BAJUSZ, E. AND MONGEAU, A.—(1963) *Rev. canad. Biol.*, **22**, 113.
*Idem*, BOIS, P. AND MONGEAU, A.—(1960) *Experientia*, **16**, 212.
MÜHLBOCK, O.—(1960) ' Steroid-Induced Tumours in Animals. Biological Activities of Steroids in Relation to Cancer '. New York (Academic Press Inc.), pp. 331–42.
*Idem* AND VAN NIE, R.—(1960) ' Hormone Dependence and Autonomy ', in ' Biological Approaches to Cancer Chemotherapy ', edited by R. J. C. Harris. London (Academic Press Inc.), pp. 277–84.
STOUT, A. P.—(1946) *Ann. Surg.*, **123**, 447.